# TSHB R75G is a founder variant and prevalent cause of low or undetectable TSH in Indian Jews

**DOI:** 10.1530/ETJ-21-0072

**Published:** 2021-11-26

**Authors:** David Shaki, Marina Eskin-Schwartz, Noam Hadar, Emily Bosin, Lior Carmon, Samuel Refetoff, Eli Hershkovitz, Ohad S Birk, Alon Haim

**Affiliations:** 1 Pediatric Endocrinology Unit, Saban Pediatric Medical Center for Israel, Beer Sheva, Israel; 2 Faculty of Health Sciences, Ben-Gurion University of the Negev, Beer Sheva, Israel; 3 Genetics Institute at Soroka University Medical Center and the Morris Kahn Laboratory of Human Genetics, National Center for Rare Diseases, at the Faculty of Health Sciences and National Institute for Biotechnology in the Negev, Ben-Gurion University of the Negev, Beer Sheva, Israel; 4 Endocrinology Lab, Soroka University Medical Center, Beer Sheva, Israel; 5 Departments of Medicine and Pediatrics and the Committee on Genetics, The University of Chicago, Chicago, Illinois, USA

**Keywords:** undetectable TSH, Indian Jews, Bene Israel, TSHB R75G

## Abstract

**Objective:**

Bi-allelic loss-of-function mutations in *TSHB*, encoding the beta subunit of thyroid-stimulating hormone (TSH), cause congenital hypothyroidism. Homozygosity for the TSHB p.R75G variant, previously described in South Asian individuals, does not alter TSH function but abrogates its detection by some immune detection-based platforms, leading to erroneous diagnosis of hyperthyroidism. We set out to identify and determine the carrier rate of the p.R75G variant among clinically euthyroid Bene Israel Indian Jews, to examine the possible founder origin of this variant worldwide, and to determine the phenotypic effects of its heterozygosity.

**Design:**

Molecular genetic studies of Bene Israel Jews and comparative studies with South Asian cohort.

**Methods:**

TSHB p.R75G variant tested by Sanger sequencing and restriction fragment length polymorphism (RFLP). Haplotype analysis in the vicinity of the *TSHB* gene performed using SNP arrays.

**Results:**

Clinically euthyroid individuals with low or undetectable TSH levels from three apparently unrelated Israeli Jewish families of Bene Israel ethnicity, originating from the Mumbai region of India, were found heterozygous or homozygous for the p.R75G TSHB variant. Extremely high carrier rate of p.R75G TSHB in Bene Israel Indian Jews (~4%) was observed. A haplotype block of 239.7 kB in the vicinity of *TSHB* shared by Bene Israel and individuals of South Asian origin was detected.

**Conclusions:**

Our findings highlight the high prevalence of the R75G TSHB variant in euthyroid Bene Israel Indian Jews, demonstrate that heterozygosity of this variant can cause erroneous detection of subnormal TSH levels, and show that R75G TSHB is an ancient founder variant, delineating shared ancestry of its carriers.

## Introduction

Thyroid-stimulating hormone (TSH) belongs to the glycoprotein hormone family, whose members harbor the shared α subunit and a unique β subunit. The TSH β subunit, encoded by human *TSHB*, is a protein of 138 amino acids, that is subsequently cleaved to result in a mature protein of 118 amino acids (1). *TSHB* mutations cause congenital non-goitrous hypothyroidism 4 (OMIM#275100), clinically presenting as profound central hypothyroidism, through loss of function mechanism.

In contrast, the previously described R75G TSHB variant (formerly referred to as R55G), a single nucleotide substitution (c.223A>G) resulting in the replacement of arginine with glycine at position 75 of the protein, does not impair the function of TSH β but rather results in a structural change that prevents recognition of TSH by some of the monoclonal antibodies used in commercial TSH immune-detection platforms (2). Using these detection platforms, TSH could not be detected in R75G homozygotes, and heterozygotes have been reported to have reduced TSH levels within the reference range. The R75G variant has been reported to be prevalent in individuals of South Asian ethnicity (2) including Pakistan and India (3).

Clinically, individuals homozygous for the R75G TSHB variant cannot be distinguished from patients with subclinical hyperthyroidism, defined biochemically as having normal serum free thyroxine (T4) and triiodothyronine (T3) concentrations in the presence of a subnormal serum level of TSH. Accordingly, falsely undetectable TSH can be easily confused with endogenous (e.g. toxic adenoma, Graves’ disease or thyroiditis) or exogenous (e.g. levothyroxine overdose) causes of hyperthyroidism/thyroid hormone excess, as well as with the presence of the immunoassay interference issues due to the existence of endogenous antibodies (4, 5).

In the current study, we describe three unrelated families of Bene Israel Indian Jewish ethnicity, with clinically euthyroid individuals having low or undetectable TSH levels and harboring the heterozygous or the homozygous R75G TSHB variant, respectively. We demonstrate the extremely high carrier rate of this founder variant among Bene Israel Indian Jews and through SNP analysis show this variant to be a founder mutation shared by South Asian populations as well as by individuals of the Bene Israel Indian Jew ethnicity, due to shared ancestry.

## Methods

### Patients

Clinical data and DNA samples were obtained following informed consent and approval of Soroka Medical Center Internal Review Board. Phenotyping was performed by senior pediatric endocrinologists and geneticists.

### Thyroid function tests

Five different Food and Drug Administration-approved immunoassays were used to determine the TSH levels (reference range 0.4–4 mU/L): ADVIA Centaur TSH-3 Ultra (TSH3-UL), ADVIA Centaur TSH (second-generation; hereafter referred to as TSH2), IMMULITE 2000 TSH, Architect TSH (Abbott Laboratories), and COBAS-Roche MODULAR E170 TSH (Roche Diagnostics). TSH was quantified using undiluted specimens according to the manufacturer’s recommendations. Free T4 (FT4) (reference range, 0.8–1.5 ng/dL) and free T3 (FT3) (reference range, 3.1–6.8 pmol/L) were measured on the ADVIA Centaur XP system according to the manufacturer’s protocols. The presence of negative inference was examined by serum dilution with TSH3-UL diluent and heterophile antibody-blocking reagent (Scantibodies Laboratory, Inc, Santee, CA, USA) according to the protocol supplied by the manufacturer (6).

### Genetic analysis

#### Genotyping

DNA was extracted from peripheral blood lymphocytes of the study subjects using the QIAamp DNA Mini Kit (Qiagen). To verify the presence of the *TSHB* (NM_000549.5), c.223A>G, p.Arg75Gly variant, Sanger sequencing was performed. Primer sequences will be provided on request.

#### Population screening

The presence of the R75G *TSHB* variant in the cohort of 70 Bene Israel Jewish individuals was examined by restriction fragment length polymorphism (RFLP) analysis. Primer sequences were 5’-AGAGGAGGGTCTCACTTTTGTC-3’ – forward and 5’-ACCACTTAAGCTCTCTAACGCC-3’ – reverse, and annealing temperature was 62°C. The mutation altered part of the PCR product sequence from 5’-CATAT*A*-3’ to 5’-CATAT*G*-3’, thereby creating restriction site for NdeI (New England Biolabs). PCR products were cut by Ndel according to the manufacturer’s instructions. RFLP products of 343 bp and 249 bp and of 592 bp were anticipated for mutant and for WT alleles, respectively.

#### Haplotyping

DNA samples from patients homozygous to the *TSHB* mutation of Pakistani, Indian, and South Asian origin (kindly provided by Prof S Refetoff, University of Chicago, Chicago, IL, USA) as well as two Bene Israeli Indian Jew samples were analyzed by SNP haplotyping. For this purpose, the Illumina Omni Express Beadchip, containing 630,000 SNPs, was used (Illumina) as previously described (7). Shared homozygous loci were found using HomozygosityMapper (8).

## Results

### Clinical findings

**Figure 1:**
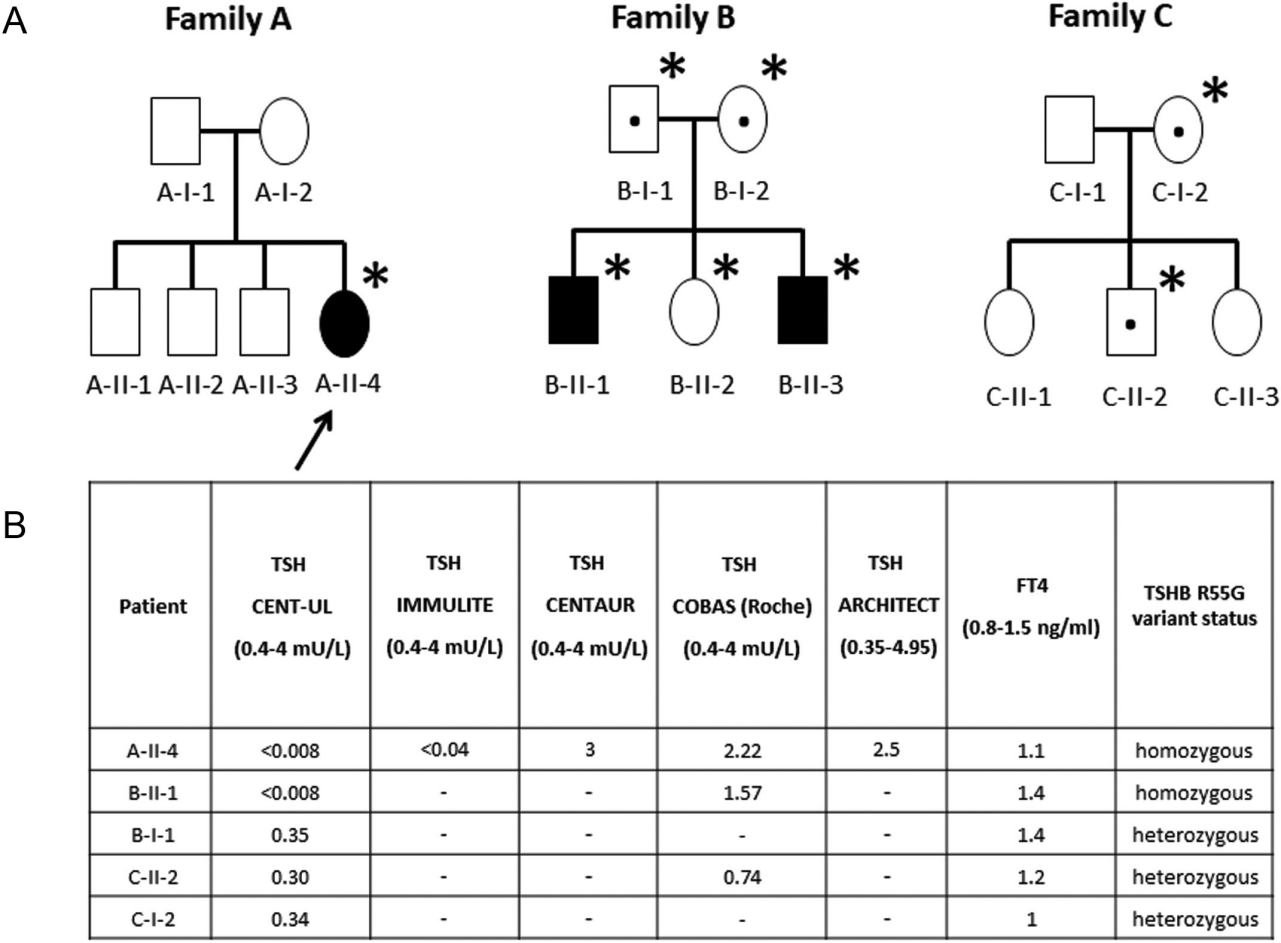
(A) Family trees of Bene Israel families included in the study: solid black symbols, individuals homozygous for the R75G TSHB variant; black dots, heterozygous carriers; asterisks, genotyped individuals. (B) TSH and FT4 levels of study subjects measured using different immunometric platforms.

The index case, a 17-year-old female of Bene Israel Indian Jewish origin (Fig. 1A and B), presented with undetectable TSH levels, following laboratory workup for hair loss using ADVIA Centaur TSH3-UL TSH platform. FT3 and FT4 levels were within the normal range. These results remained unchanged following three additional TSH measurements. The patient reported irregular menstrual cycles, mild and infrequent palpitations, and slightly increased appetite. Her weight was stable. She had standard nutrition and denied consuming food supplements and family history of thyroid disorders. Her physical examination revealed normal pulse rate and blood pressure, absence of goiter, exophthalmos, muscle weakness, or tremor. Anti-TPO (anti-thyroid peroxidase) and anti-TG (anti-thyroglobulin) antibodies were negative. During the following months, the patient has lost ~2 kg despite having increased appetite. She was described by her family members as very nervous. Thyroid function test remained unchanged. Thyroid technetium scan was performed and was qualitatively interpreted to show diffusely increased uptake. Serum dilution testing to detect possible anti-streptavidin antibody immunoassay interference was negative. Testing for the presence of the heterophilic antibodies in serum was negative as well. At that point, the diagnosis of Graves’ disease was made and low dose mercaptizol treatment was initiated. Following 1 year of treatment, the patient reported improvement in her mood and returned to her baseline weight.

Repeated thyroid function tests revealed no change in the TSH levels, suggesting the presence of undetectable TSH. Blood samples were sent for thyroid function testing on four additional platforms, including the second-generation ADVIA Centaur TSH, IMMULITE 2000 TSH, Architect TSH, and COBAS-Roche MODULAR E170 TSH. In all but IMMULITE 2000 TSH and ADVIA Centaur TSH, the TSH, FT4, and FT3 levels were detected to be within normal range. Mercaptizol was discontinued. Thyrotropin receptor antibodies assay was completed and was found to be negative.

### Genetic analysis

Sanger sequencing revealed the patient to be homozygous for the R75G TSHB variant.

Following the index case, two additional familial cases of clinically asymptomatic Bene Israel Indian Jews having low or undetectable TSH levels, using ADVIA Centaur TSH3-UL TSH detection platform, have been encountered. All had FT4 in the normal range and were found to be heterozygous or homozygous for the R75G TSHB variant, respectively (Fig. 1A and B).

To estimate the frequency of the R75G TSHB variant carrier rate among Bene Israel Indian Jews, 70 additional DNA samples of individuals of this ethnicity were analyzed using RFLP. Of these, three samples were found to be heterozygous for this variant, suggesting an overall ~2% allele frequency and ~4% carrier rate in this cohort.

**Figure 2:**
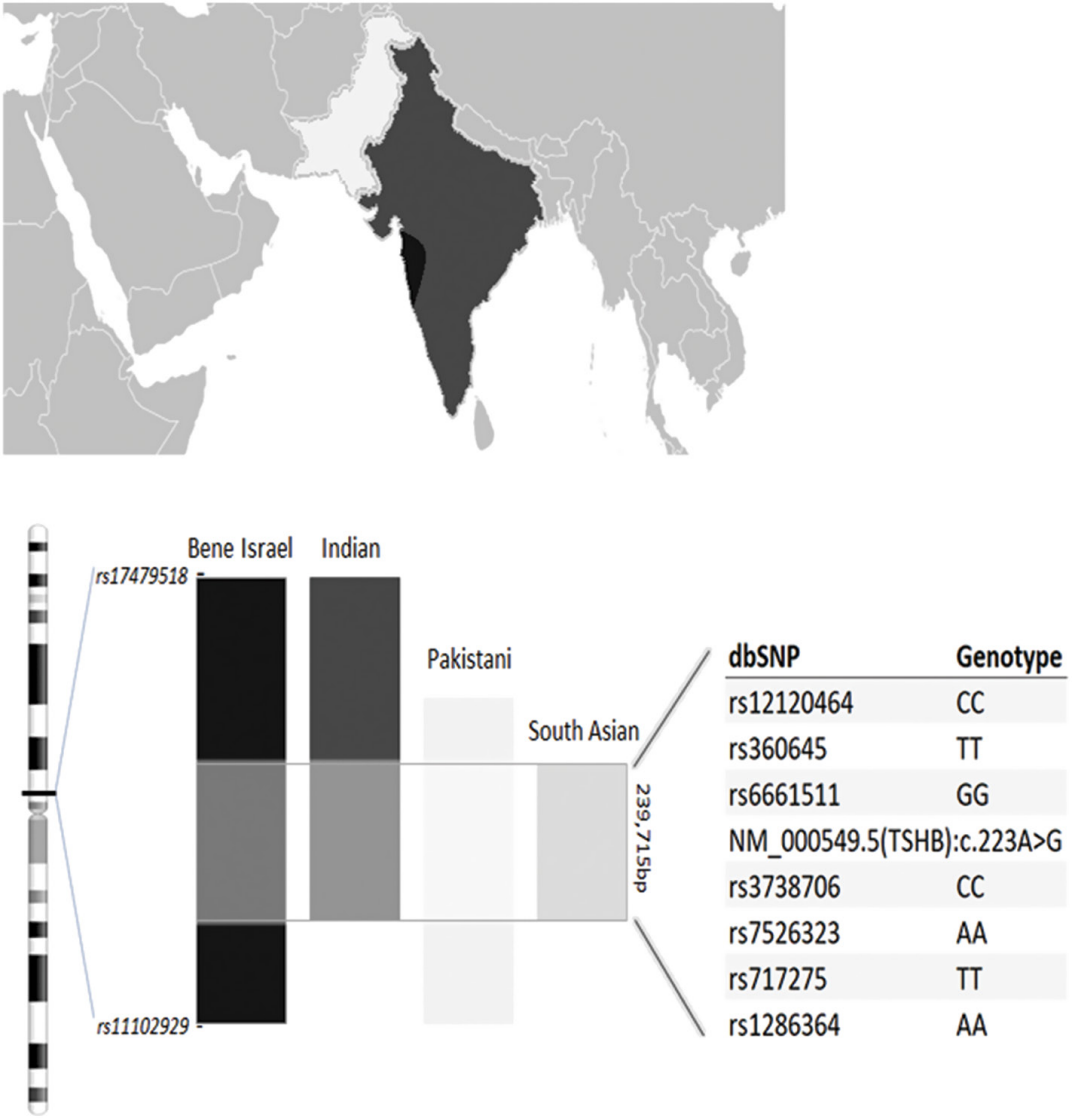
SNP haplotyping of the DNA samples from patients homozygous for the R75G TSHB variant of Pakistani, Non-Jew Indian, South Asian, and Bene Israeli Indian Jewish origin revealed the common 239.7 kB haplotype block, shared by all the examined samples.

SNP haplotyping of the DNA samples from patients homozygous for the R75G TSHB variant of Pakistani, Non-Jew Indian, South Asian, and Bene Israeli Indian Jewish ancestry revealed the R75G TSHB variant to reside within a haplotype block of 239.7 kB, shared by all the examined samples. These results suggest the R75G TSHB variant to represent a founder variant, shared by Bene Israel Indian Jews and the South Asian non-Jewish population (Fig. 2).

## Discussion

TSH level determination is a widely used laboratory test, crucial for correct diagnosis of thyroid dysfunction disorders (9, 10). Immunodetection is nowadays considered the method of choice to detect TSH due to its high sensitivity (11). However, in rare cases, false results are obtained. Some are caused by the presence of macro TSH in the sample, while others are attributed to the presence of antibodies reactive with the immunoassay ingredients (4, 5). In the commercially available TSH immunometric platforms, TSH is detected by the use of two monoclonal antibodies, the solid phase antibody and a detection antibody, usually targeting the beta subunit and the alpha–beta interface of the TSH (1). Since most platforms utilize monoclonal antibodies, they vary in their ability to detect mutated circulating TSH (12, 13, 14, 15, 16).

Accordingly, in recent years, it became apparent that the ability to immune-detect TSH does not necessarily correlate with its functional state. Thus, mutations in the *TSHB* gene impairing TSH function but still allowing it to be detected have been reported (17). The opposite also holds true, as the R75G TSHB missense variant, reported not to alter TSH function, has been reported to impair TSH detection by a number of the widely used immune-detection reagents manufactured by Siemens, including ADVIA Centaur TSH3-UL, Immunlite 2000, Dimension Vista, and Dimension (1, 2).

These data are of importance since the heterozygous carrier rate of the R75G TSHB variant was found to be high among subjects originating from South Asia (including Pakistan and India), being five-fold higher compared to the general population (1000 Genomes database Minor Allele Frequency (MAF) of 0.012 for South Asians) (1).

Medical record review of a cohort of individuals homozygous for the R75G TSHB variant revealed that because of the falsely undetectable TSH levels many of them have been incorrectly diagnosed with hyperthyroidism and treated (2), similar to our index case.

Furthermore, falsely undetectable TSH levels can also lead to erroneous diagnosis of central hypothyroidism in affected individuals with primary hypothyroidism.

Heterozygotes have been previously reported to have reduced (yet within the reference range) TSH levels using Siemens Immulite 2000 and Siemens Centaur TSH3 Ultra (1). Interestingly, in our cohort, individuals heterozygous for the R75G TSHB variant had below the normal range TSH levels using the same TSH detection reagents as previously described (1). This could possibly reflect the interindividual variability of the normal TSH levels, and suggest that the presence of the R75G TSHB variant could be erroneously interpreted as subclinical hyperthyroidism in heterozygous carriers as well.

Our data suggest an extremely high carrier rate of the heterozygous R75G TSHB variant (MAF of ~2%) in the Israeli inbred Jewish community of Bene Israel.

The Bene Israel Jewish community originates from a cohort of Jews in India, originally living in the Konkan area, who settled in the 19th century in Mumbai and in other cities of West India, as well as in Karachi of Pakistan. In 1948, the Bene Israel Jewish community of India comprised 20,000 people. Since then, most of them immigrated to Israel. The genetic background of this community has been extensively studied, revealing Bene Israel to represent the genetic admixture of both Indian and Middle East and Jewish ancestries (18). The contemporary Indian populations carry the genetic background of both Ancestral North Indians (ANI), genetically related to west Eurasians, and Ancestral South Indians (ASI) (19, 20). Notably, some Pakistani populations (geographically bordering North India) were also reported to represent the ANI–ASI admixture (19, 20) similar to Indians, and some were found to carry genetic components similar to Jewish and Middle-Eastern populations (18). These data are consistent with our haplotype analysis results, revealing the shared haplotype block (including the *TSHB* gene) between Bene Israeli Jews and the Pakistani and Indian subjects and suggesting the R75G TSHB variant to be a common founder variant shared by these populations.

In summary, our findings demonstrate the R75G TSHB to be an ancient founder mutation due to shared ancestry of its carriers, highlight its presence and high prevalence in Bene Israel Jews, and show that heterozygosity of this variant can cause low measurable TSH levels and need to be included in the differential diagnosis of subclinical hyperthyroidism.

## Declaration of interest

The authors declare that there is no conflict of interest that could be perceived as prejudicing the impartiality of the research reported.

## Funding

The study was supported by the Morris Kahn Family Foundation, the National Knowledge Center for Rare/Orphan Diseases of the Israel Ministry of Science, Technology and Space at Ben Gurion University, and the Israel Science Foundation (grant no. 2034/18), all awarded to O S B. The study was supported in part by grant DK15070 from the National Institutes of Health, USA awarded to S R.

## Ethics declaration

The participants of this study provided written informed consent according to a protocol approved by the Soroka Medical Center institutional review board and by the Israel National Committee for Human Genetic Studies, in adherence with the Helsinki principles.

## Data availability

Will be available upon request.

## Author contribution statement

D S was responsible for screening potentially eligible subjects, extracting and analyzing data, interpreting clinical results, and participated in writing the manuscript. M E S was responsible for designing the genetic analysis, analyzing molecular data, and writing the manuscript. N H and E B conducted the molecular lab work (genetics and endocrinology respectively). L C helped screening potentially eligible subjects. S R provided DNA samples of Asian individuals for SNP array analysis and provided feedback on the manuscript. E H, O S B, and A H participated in study design, provided funding, and feedback on the manuscript. O S B and A H contributed equally.
